# A statistical model that predicts the length from the left subclavian artery to the celiac axis; towards accurate intra aortic balloon sizing

**DOI:** 10.1186/1749-8090-6-95

**Published:** 2011-08-09

**Authors:** Haralabos Parissis, Alan Soo, Michalis Leotsinidis, Dimitrios Dougenis

**Affiliations:** 1 Department of Cardiothoracic Surgery, Royal Victoria Hospital, Belfast, BT12 6BA, UK & Northern Ireland; 2 Department of Statistics & Epedemiology University of Patras Medical School, Patras, 26504, Greece; 3 Cardiothoracic Department University of Patras Medical School, Patras, 26504, Greece

## Abstract

**Background:**

Ideally the length of the Intraaortic balloon membrane (22-27.5 cm) should match to the distance from the left subclavian artery (LSA) to the celiac axis (CA), (LSA - CA). By being able to estimate this distance, better guidance regarding IABP sizing could be recommended.

**Methods:**

Internal aortic lengths and demographic values were collected from a series of 40 cadavers during autopsy. External somatometric measurements were also obtained.

There were 23 males and 17 females. The mean age was 73.1+/-13.11 years, weight 56.75+/-12.51 kg and the height 166+/-9.81 cm.

**Results:**

Multiple regression analysis revealed the following predictor variables (R2 > 0.70) for estimating the length from LSA to CA: height (standardized coefficient (SRC) = 0.37, p = 0.004), age (SRC = 0.35, p < 0.001), sex (SRC = 0.21, p = 0.088) and the distance from the jugular notch to trans-pyloric plane (SRC = 0.61, p < 0.001).

Recommendations: If LSA-CA < 21.9 cm use 34 cc IABP & if LSA-CA > 26.3 cm use 50 cc IABP. However if LSA-CA = 21.9- 26.3 cm use 40 cc, but be aware that it could be "aortic length-balloon membrane length" mismatching.

**Conclusions:**

Routinely, IABP size selection is being dictated by the patient's height. Inevitably, this leads to pitfalls. We reported a mathematical model of accurate intraaortic balloon sizing, which is easy to be applied and has a high predictive value.

## Introduction

The selection of the intraaortic balloon pump (IABP) in adults has been mainly limited to the use of 40 cc and occasionally 34 cc balloon volume, with a membrane length that varies among manufacturers from 22 to 27.5 cm and an inflated diameter 15 - 18 mm (Table 1).

**Table 1:** IABP sizes

Volume	Membrane length	Inflated diameter
34 **cc**	21.9 **(cm)**	14.7 **(cm)**
40	26.3	15 -16.2
50	27.5	18.3

Although in a number of clinical cases the 40 cc volume is sufficient, it shall be noted that an increased balloon volume could potentially contributes to morbidity from vascular events and a reduced balloon volume could decrease the beneficial hemodynamic effects for the heart. There is a causal relashionship between the patient's height and balloon trauma. According to Cox et al [1] the balloon size shall be adjusted depending on the patient's height.

The ideal balloon for any patient shall be placed at an ideal position, which equals in length the distance from the left subclavian artery to the celiac axis, 90-95% of the diameter of the descending aorta and equals in volume the blood volume inside the aorta lumen at all given time [2].^.^

Modern treatment guidelines (Table 2) suggest for patients up to 162 cm high the use of 34 cc balloons, for patients 162-182 cm high a 40 cc balloon and for patients over 182 cm high the use of 50 cc balloons. The problem arises when the length of the balloon membrane is greater than the length from the left subclavian artery to the celiac axis. Renal arteries and visceral circulation may potentially be occluded at each inflation of the balloon.

**Table 2:** The current recommendations regarding IABP sizing

34 cc	40 cc	50 cc
147-162 cm	162-182 cm	> 182 cm
BSA < 1.8	BSA > 1.8	

This paper is a pilot study aiming to predict the length of several anatomical lengths in the descending aorta and by extension to lead to the optimum IABP size selection for every individual patient.

The main question aims to identify an equation that can calculate the length from the take off of the left subclavian artery to the origin of the celiac axis. The length of the balloon membrane in association with the distance from the left subclavian artery to the take off of the celiac axis will be used as a possible guide to the optimum sizing of the IABP.

## Material and methods

Measurements were carried out from a series of 40 cadavers during autopsy. Twenty-three were male cadavers and 17 female. The mean age was 73.1+/-13.11 years, mean height was 166+/-9.81 cm and mean weight was 56.75+/-12.51 kg.

**Figure 1:**
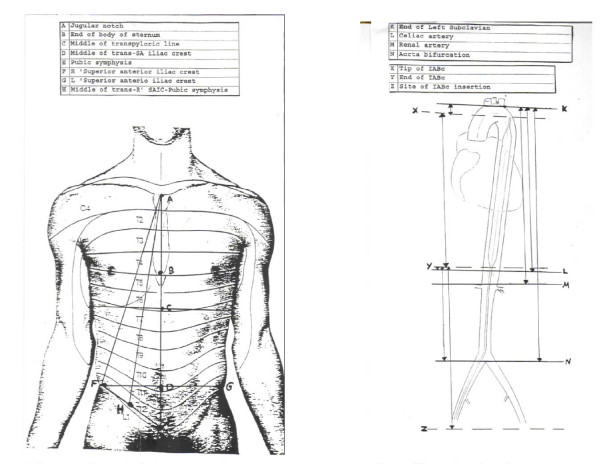
**1a) External measurements of easily obtainable anatomical landmarks and 1b) internal aortic measurements**.

**Figure 2:**
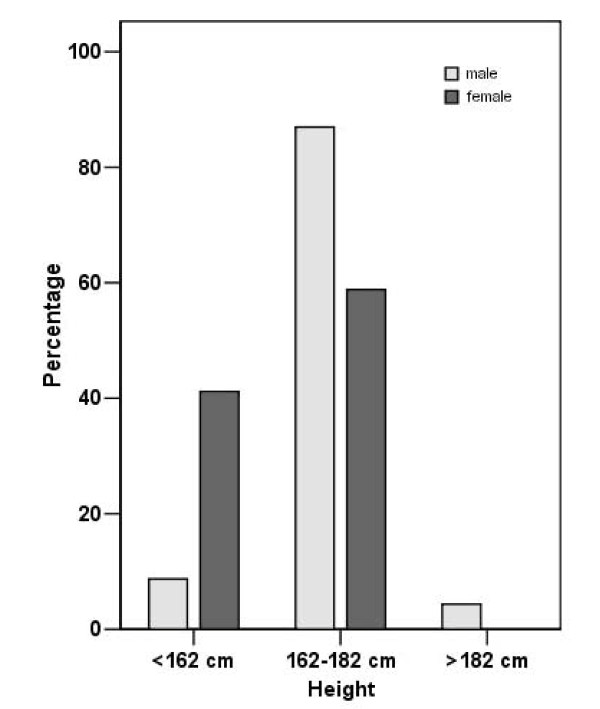
**Graphic representation of the height distribution between genders**.

External somatometric measurements were obtained before autopsy (Figure 1a). With intact aortic length, the internal measurements were carried out in situ (Figure 2a). The distance between the origin of the left subclavian artery and the insertion point of the catheter was measured by calculating the length of a guide wire that was introduced to the aortic lumen, from a point 1 cm below the middle inguinal region to the orifice of the subclavian artery (cross-validated via digital palpation). All other internal measurements were carried out after the anatomical aortic dissection during autopsy (see Table 3, and Table 4).

**Table 3:** Row data and measurements depicted in Figure 1

Demographics						External Lengths (cm) Diameters								Internal Lengths				Depth
**Pt**	**Hght**	**Wght**	**Age**	**SEX**	**BSA**	**Meas1**	**Meas2**	**Meas3**	**Meas4**	**Meas5**	**Dthrax**	**Dren**	**Dbifrc**	**LSA-CA**	**LSA-Ren**	**LSA-Bifr**	**LSA-Insr**	**Dpth**

**1**	168	55	89	m	1.62	26	45	24.25	43.25	49	30	29	25	22.5	27.5	37.5	53	2.5
**2**	178	71	76	m	1.88	29.5	53.1	27.4	51	58.2	29	28	24	26	27.6	36	52	2.3
**3**	148	47	84	f	1.38	25.5	44.5	23.25	42.25	49	31	26	21	22.5	25	32.5	50.5	2.2
**4**	143.5	41	79	f	1.28	21.5	44	19.45	41.95	49.4	28	23	20	19.5	21.4	31	48.5	2.4
**5**	165	55	89	f	1.60	26	44	24.05	42.55	50.5	27	25	20	23	26	36.5	53.5	2.1
**6**	163	46	34	f	1.47	26.5	49	24.6	47.6	51	21	20	17	20.5	23.4	32.2	51	2.1
**7**	153	63	52	f	1.60	27.5	50	25.3	47.8	53	24	21	18	24	27	34	49.5	2.9
**8**	185	50	77	m	1.67	27	47	24.6	44.6	51.5	32	30	26	27	29.5	41	57.8	1.8
**9**	165	50	73	m	1.53	28.5	50.7	26.65	48.85	52	23	21	17	23	25	38	51	2.1
**10**	163	41	56	f	1.40	22	40	20.35	38.35	42.3	18	17	13	20	23	33	47	1.8
**11**	173	45	83	f	1.52	26.5	47	24.75	45.25	48	23	21	18	26	29	41	56.5	2.0
**12**	165	66	68	f	1.73	25	43	23.5	41.5	45.6	27	24	19	24.5	28	38.5	52.6	2.5
**13**	173	63	60	f	1.75	26	46.5	24	44.5	49.5	29	26	21	23	26.5	37.6	53.2	2.4
**14**	175	64	58	f	1.78	26.5	50.5	24.25	48.25	53.8	28.5	27	20	24.5	27.5	37.7	54.3	2.6
**15**	170	55	74	f	1.63	26	48	24	46	52.5	26	24.5	19.5	25	28.7	36.6	53.6	2.3
**16**	175	63	74	m	1.77	27.5	49	25	46.5	53.7	24	22.5	18	22	26.8	38.8	51.5	1.8
**17**	173	64	94	m	1.76	26.5	46	24.5	44	49.5	25.5	23	17.5	23.5	25.5	36.2	50.7	2.0
**18**	180	64	76	m	1.82	25	44.5	23	42.5	50.6	24	22.5	18	24	27.8	38.3	56.5	1.6
**19**	153	54	42	f	1.50	25.5	46	23.75	44.25	49.8	26	24.5	19	20.5	23.3	31.5	47.8	2.1
**20**	173	84	70	m	1.98	31	52	28.1	49.1	56.2	28	27	19.5	29	31.5	40.7	55.9	3.1
**21**	179	78	71	m	1.97	29.5	51	27.25	48.75	56.7	29	27.5	20.5	28	32	42.9	58.1	2.3
**22**	180	98	74	m	2.18	31	51.5	28.5	49	55.8	28.5	26.5	18.5	27	31.8	43.7	59.6	3.3
**23**	178	55	73	m	1.69	27.5	48.5	24.75	45.75	53.9	24	23	17	25.5	29.7	41.8	58.7	2.1
**24**	163	64	83	m	1.69	28.5	49	25.75	46.25	52.8	27	25.5	18.5	23	26.9	35.8	52.6	2.6
**25**	163	43	75	f	1.43	25.5	44.5	23.85	42.85	48.2	23	21	16.5	23	26.5	35.7	50.8	1.7
**26**	158	40	57	m	1.35	24.5	42	22.75	40.25	45.6	22	20.5	14.5	20	23.6	30.9	46.6	1.8
**27**	178	72	69	m	1.89	27	46.5	24.75	44.25	51.2	24	21	15.5	24	28.3	39	56.8	1.9
**28**	163	56	63	m	1.60	26	45	24.25	43.25	49.2	23.5	22	16	23	26.8	36.1	50.4	2.0
**29**	170	46	84	m	1.51	25.5	43.5	23.25	41.25	47.4	25	23.5	17	24	28.1	38.8	53.9	1.6
**30**	180	57	73	m	1.73	27	49	24.25	46.25	54.6	27	25	18.5	25	29.6	42.3	57.7	1.7
**31**	155	55	76	f	1.53	24.5	45.5	22.25	43.25	48.7	24.5	22.5	19	23	26.2	33.1	48.3	1.9
**32**	156	54	71	f	1.52	24	43.5	21.75	41.25	46.5	23.5	22.5	17.5	22	25.3	32.4	49.1	1.8
**33**	153	42	93	f	1.35	21.5	42.4	19.75	40.65	46.2	22	20.5	15.5	21.5	24.8	31.3	47.9	1.5
**34**	165	55	87	m	1.60	26.5	49.5	24	47	52.9	27.5	25	19.5	26	28.5	37.2	51.8	1.8
**35**	175	66	73	m	1.80	28	47	25.25	44.25	52.1	26	24.5	18	24	28.9	38.1	54.1	1.5
**36**	163	40	90	m	1.38	26.5	44	24.25	41.75	48.2	24.5	22	17.5	26	28.1	37.4	50.9	1.3
**37**	163	62	88	m	1.67	25	46.5	22.75	44.25	49.6	28.5	27	20	24	27.8	36.9	51.6	1.9
**38**	165	49	69	f	1.52	24.5	43.5	22.75	41.75	47.7	26	24.5	18.5	23	27.1	36.1	50.2	1.7
**39**	165	47	80	m	1.50	25	44	23	42	48.4	25.5	23.5	16.5	23	26.9	35.3	51.1	1.5
**40**	158	50	67	m	1.49	25	45.5	22.75	43.25	48	26	24.5	17.5	22	25.6	34.2	49.9	1.7

(The Trans-pyloric plane is located halfway between the jugular notch and the upper border of the pubic symphysis);

**Table 4:** Definitions of the variables analysed in table 3 (eg. Measurements, Diameters, lengths between various aortic branches)

**Measurement 1**	from the jugular notch to the trans-pyloric plane	**LSA-CA**	from the left subclavian take off to the celiac axis
**Measurement 2**	from the jugular notch to the anterior superior iliac spine	**LSA-Renal**	from the left subclavian take off to the renal artery
**Measurement 3**	from the middle point between the jugular notch and the sternal angle to the trans-pyloric plane	**LSA-Bifur**	from the left subclavian take off to the aortic bifurcation
**Measurement 4**	from the middle point between the jugular notch and the sternal angle to the anterior superior iliac spine.	**LSA-Inser**	from the left subclavian artery take off to the IAB insertion 2 cm below the mid-inguinal ligament
**Measurement 5**	from the jugular notch to the middle point in the line between the anterior superior iliac spine and symphysis pubis	**Depth**	the depth of the femoral artery from the surface of the skin, 2 cm below the mid-inguinal ligament
**Dthorax**	Aortic diameter (at the middle of the thorax)		
**Drenal**	Aortic diameter (at a renal level)		
**Dbifurc**	Aortic diameter (bifurcation level)		

Our Institutional Review Board approved this study. Individual consents were obtained for all cadaveric examinations and entry into the database.

### Statistical Processing

Statistical analysis was performed with SPSS 17.0 statistical software (SPSS Inc., Chicago, IL, USA). Unpaired student t-test was applied to compare the various measurements between the two genders. Pearson *r *correlation coefficients were calculated to identify association between variables. Multiple linear regression models were used to predict the sizes of the aorta. The validation of the prediction for the balloon sizing was performed using discriminant analysis. The Cohen's Kappa test was employed to assess the agreement between the selections of the balloon size based on the results of the categorization by using the discriminant analysis model or using the "traditional" categorization (sizing as it is derived by the height of patients). The statistical significant level was set at 0.05

## Results

There have been differences between the two genders with regard to the aortic lengths, with greater differences observed in the distance from the left subclavian artery to the aortic bifurcation. However there have been no differences in the comparison of the internal diameters, between the two genders. (Table 5)

**Table 5:** Gender comparison of external measurements, aortic lengths and diameters

	male (n = 23)		female (n = 17)	
	mean	S.D	mean	S.D
Height(cm)*	171.09	7.98	160.97	9.20
Weight*	60.30	13.93	51.94	8.51
BSA*	1.70	0.20	1.28	0.14
BMI	20.50	3.93	20.10	3.25
Age	76.57	9.15	68.41	16.24
Measur1*	27.11	1.88	24.97	1.80
Measur2*	47.38	3.03	45.41	2.83
Measur3*	24.83	1.72	23.04	1.76
Measur4	45.10	2.88	43.53	2.75
Measur5*	51.61	3.31	48.92	2.88
Dthorax	26.24	2.53	25.15	3.25
Drenal	24.52	2.67	22.94	2.62
Dbifurc	18.70	2.88	18.38	2.06
LSA-CA*	24.41	2.14	22.68	1.84
LSA-Rena*	27.99	2.11	25.81	2.12
LSA-Bifur*	38.13	3.00	34.75	2.95
LSA-Inser*	53.57	3.43	50.84	2.71
Depth	2.01	0.49	2.12	0.37

*p < 0.05 (t-test)

The Table 6 and Figure 2 shows the rates for both genders, depending on the distribution of height. One can see that the majority of males is 162-182 cm high, whereas females are almost equally divided between (< 162 cm) and (162-182 cm).

**Table 6:** Height distribution between genders

Height	male (%)	fem (%)
< 162 cm	8.7	41.2
162-182 cm	87.0	58.8
> 182 cm	4.3	

With regards to the differences in the aortic diameter above and below the renal arteries in patients < 162 cm, 162-182 cm and > 182 cm, a weak association is observed (r = 0.29, p = 0.07). In addition, this difference in the aortic diameter shows no variance among the three groups or the two genders.

The prediction of intra-aortic diameters was poor (R^2 ^< 0.30). However the prediction of the internal measurements by using multiple regression analysis was satisfactory (R^2 ^> 0.70) as per Table 7.

**Table 7:** Multiple regression model

	Predictor variables	URC	SRC	p*
LSA-CA	Height	0.08	0.37	0.004
R^2 ^= 0.71	Age	0.06	0.35	< 0.001
	^Ŧ^Gender	0.91	0.21	0.088
	Measurement 1	0.63	0.61	< 0.001
	Constant	-12.05	-	0.021

LSA-RENAL	Height	0.11	0.45	< 0.001
R^2 ^= 0.70	Age	0.04	0.24	0.001
	Measurement 1	0.48	0.43	< 0.001
	Constant	-6.90	-	0.077

LSA-BIF	Height	0.23	0.66	< 0.001
R^2 ^= 0.81	Age	0.05	0.19	0.017
	Measurement 1	0.46	0.28	0.005
	Constant	-16.99	-	< 0.001

LSA-INS	Height	0.25	0.71	< 0.001
R^2 ^= 0.71	Measurement 5	0.23	0.23	0.029
	Constant	-0.63	-	0.029

(R^2 ^> 0.70)

URC: Unstandardized coefficient

SRC: Standardized coefficient

*Only the coefficients with a p < 0.10 are presented

^Ŧ^male = 1, female = 2

The selection of the balloon size in the traditional methods, depending on the height, shows relatively low consistency (although statistically significant) to the size that would have been selected if the actual length of the internal dimensions of the descending aorta was known. (Consistency test K = 0.38, p = 0.003).

On the contrary, the selection of the balloon size based on the results of the categorization by using the discriminant analysis model, shows impressive consistency with the sizes that would have been selected based on the actual aortic length (K = 1, p < 0.001).

In order to apply the results of the regression model to predict the length from the left Subclavian artery take off to the Celiac axis (LSA-CA) the following equation is formed:

If LSA-CA < 21.9 cm use 34 cc IABP, LSA-CA = 21.9- 26.3 cm use 40 cc (may be "aortic length-balloon membrane length" mismatching) & if LSA-CA > 26.3 cm use 50 cc IABP.

As far as the depth of the femoral artery is concerned at the inguinal plane, there are no differences between the two genders (t Test) but there is an increased association between weight and depth (r = 0.49, p = 0.002).

## Discussion

The efficiency of the IABP depends on multiple factors, which include: The ability of the ejection system to trace electrical stimulation events and transfer the gas during every cardiac cycle, the patient's hemodynamic status, the anatomical position of the balloon and lastly the volume of the selected balloon.

The variables that affect the size and duration of the IABP inflation with gas and consequently the diastolic increase in pressure, were first published 20 years ago, in an innovative study by Weber et al [3]. They reported that the arterial pressure has direct impact on the balloon performance and that the beneficial effects of the IABP reduces when arterial pressure increases.

The heart rate also affects the balloon functioning. The faster the heart rate, the less the balloon inflation time and the diastolic augmentation.

The relation between the aortic pressure and the volume of the aorta reflects the aortic elasticity and the reduced aortic elasticity means reduced diastolic augmentation.

The closer the IABP to the aortic valve, the higher is the incremented diastolic augmentation [4].

The effect of the IABP diameter to the patient's hemodynamics was studied by Weber, Janicki, and Walker [3]. They concluded that for any given arterial pressure or aortic size, the greatest augmentation in mean aortic diastolic pressure was achieved with complete occlusion. It is obvious that in 100% occlusion of the aortic lumen, a lesion to the aortic wall and erythrocytes could possibly occur and thus the ideal convergence is thought to be approximetly 90 to 95%.

The balloon volume shall be at least 50% of the stroke volume. In a hypothetical patient with poor cardiac index, the rate of the diastolic incrementation to support an insufficient heart is limited because of the fact that the stroke volume in the given patient is low and thus the ideal balloon volume shall be low. In the course of improvement of the hypothetical s patient s hemodynamics and the more the stroke volume improves, which means that the volume of blood transferred within the aorta at every stroke increases, then the balloon volume of the IABP, necessary to maintain the same level of diastolic augmentation, increments. This fact may suggest that during a period of time from the balloon implantation till the successful removal of the balloon, the performance of corrective interventions to the balloon volume by intervals, based on the patient's clinical status, may be the proper method in order to provide ideal diastolic augmentation, both for the insufficient heart and the heart that shows improvement.

To summarize up to now, the optimum balloon sizing shall be accompanied by adjustments of the balloon size and volume, which shall correspond to the different hemodynamic scenarios.

The equation to calculate the ideal balloon size for every individual patient, shall take into account the patient's hemodynamic state (Arterial pressure, Heart supply, pulses/min) as well as the measurements of the aortic length and diameter from the subclavian artery take off to the celiac axis.

Wiekel et al [5] after studying the aortographies of 169 patients, concluded that the corrected mid-thoracic diameters varied in size from 16 to 30 mm with 90% of the patients having a diameter over 19 mm. This has been possibly taken into consideration by the manufacturers and thus the balloon diastolic diameter is between 14.7 mm and 18.5 mm.

We reached the same conclusions in our measurements regarding the mid-thoracic diameters.

Paulin et al [6] suggested that the aorta's size is related to the patient's height, age and weight. After studying a series of aortic diameters, he suggested that the ascending aorta has a relatively fixed lumen, which varies from 22 to 38 mm in adults. The descending aorta has a slightly smaller lumen than the ascending aorta. In addition, the aorta's diameter narrows down after the renal artery take off.

In our measurements regarding the correlation between the difference of the aortic diameter above and below the renal arteries and the patients' height, a weak association resulted. In addition, this difference in the aortic diameter shows no variance between the two genders.

The balloon distal edge shall be "proximal" to the renal arteries' take off, so as to avoid occluding the renal arterial circulation. Swartz et al [7] studied the arterial dysfunction of the kidneys as a result of the position of the IABP on the take off of the renal arteries. He concluded that such a position of the balloon causes renal ischemia.

In our paper it became apparent that the female population consists of smaller dimensions of skeleton. The mean LSA-CA distance was found to be 22.68+/-1.84. In such a population, even a 34 cc IAB would lie below the take off of the renal arteries, with subsequent «aortic-device mismatch» and negative consequences.

During our study the traditional balloon sizing, based only on the patients height, shows relatively low consistency to the size that would have been selected if the actual length of the internal dimensions of the descending aorta was known. A report by Rastan et al [8] using CT scans identified IABP malpositions to be a common finding. Anatomic to balloon length mishmatch was found in 68.2% of the cases, with subsequently severe adverse effects.

Yosioka et al [9] reported that aortic-balloon mismatch could cause abdominal arterial branch obstruction. However, clinical reports of intra-abdominal ischemia due to anatomic-to-device length mismatch are limited [10,11]. Furthermore, Cho et al [12] has published in a CT study a 29% rate of renal artery compromise in patient heights of 163 to 183 cm when using a 40-mL balloon size, especially in patients with a small stature. In our study, we confirm by enlarge, these findings: there is only a varying correlation between height and aortic length, which is influenced by age, sex, and possibly ethnic differences [12].

According to Alexander et al [13] the rate of the patients over 70 years old that undergo heart interventions, has raised from 7-9% in the 1980's to over 30% in the 2000's. The number of women in need of cardiac interventions has increased from 31% in 1982 to over 40% today. Moreover, a 3-5% of the patients currently live their eighth decade. Sisto et al [14] mention that an increased number of patients living their eighth decade and who are in need of an IABP support.

Taking into account that that mean age of patients that need a heart operation rises (and the age increases the risk of aortic atherosclerotic disease) and the increasing rate of the women patients population (having smaller aortic diameter) which need an IABP, one would conclude that a more meticulous size selection is required under such clinical conditions, otherwise the aortic environment could be hiding potential risks. Moreover, Wolvek et al [15] reported that 35.7% of female patients fall into the category of less than 62 in. where the aortic diameter narrows down from 20 mm over the renal arteries to 13 mm below the renal arteries. In such patients, a large IABP size is problematic since 5-6 cm of the IABP fills the "normally narrowed" part of the abdominal aorta (aortic-device mismatch).

The population that we studied was 72.8% male and 27.2% female. The average height of women was 160.97 cm. A 41.2% were less than 162 cm high. Contrary to other studies, our measurements did not reveal a great aortic physiological narrowing of the aorta below the renal arteries.

The aortic measurements in our study are related to the height of the patients and other somatometric measurements (age, sex, external measurement of the thoracic cage). Neither weight nor BSA affect the aortic length or diameter.

In our measurements, the depth of the femoral artery at the inguinal region did not differ between the two genders, though it was proportionate to the body weight.

The possible limitations of the study are related to the relative small number of subjects studied, the fact that most aortas were not atheromatic, so no corkscrew effects or angulations were present and lastly the relative small number of patients over 182 cm high.

Concluding, the guidelines according to which the appropriate size of the intra-aortic balloons are selected, are based on the estmation of the LSA-CA taking into consideration the patients height, age, sex and the easily obtainable distance from the jugular notch to «halfway between the jugular notch and the upper border of the pubic symphysis»; so if LSA-CA is < 21.09 cm use 34 cc IABP, 21.9-26.3 cm use 40 cc, but prepare for «aortic-device» mismatching and if > 26.3 cm use 50 cc.

## Competing interests

The authors declare that they have no competing interests.

## Authors' contributions

HP conceived of the idea, carried out all the measurements and wrote the manuscript. AS helped in organizing the data and also helped in the process of writing the manuscript. ML carried out the statistical analysis. DD supervised the project and offer valuable points and ideas. All authors read and approved the final manuscript.
